# Effect of Narrow-Band Ultraviolet B Phototherapy and Methotrexate on MicroRNA (146a) Levels in Blood of Psoriatic Patients

**DOI:** 10.1155/2015/145769

**Published:** 2015-10-29

**Authors:** Asmaa M. Ele-Refaei, Fatma M. El-Esawy

**Affiliations:** Dermatology & Andrology Department, Faculty of Medicine, Benha University, Benha, Egypt

## Abstract

*Background*. Recently, some miRNAs have been proven to show aberrant expression in psoriasis and play a role in the pathogenesis of the disease.* Objective*. To find out whether NB-UVB or methotrexate treatment affects whole blood levels of human miRNA (146a) in patients with psoriasis and demonstrate its correlation with disease severity.* Methods*. Blood samples were obtained from healthy control and from psoriatic patients before and 12 weeks after treatment with NB-UVB, methotrexate. Quantification of human miRNA (146a) by Real Time PCR (RT-PCR).* Results*. Blood human miRNA (146a) levels were higher in patients with psoriasis than those in healthy controls (*P* = 0.001); it had no significant positive relation with PASI scores in patients (*r* = 0.2, *P* = 0.107). Real Time PCR showed that, after 12 weeks of treatment with NB-UVB phototherapy or treatment with methotrexate, there was significantly decreased level of miR146a (*P* = 0.001; *P* = 0.002, resp.).* Conclusion*. The expression of miRNA146a is increased in whole blood samples from psoriasis patients, so we can evaluate its possibility to work as a future therapeutic objective in the treatment of psoriasis. With these markers, it is able to screen therapeutics effect or changes to a further aggressive treatment for psoriasis.

## 1. Introduction

Psoriasis is a common skin disorder affecting about 3% of the world population. It is characterized by relapsing skin lesions displaying epidermal hyperplasia, an inflammatory infiltrate, and angiogenesis. The inflammatory reaction is believed to be largely the result of an interaction between innate immunity (mediated by antigen-presenting cells and natural killer T lymphocytes) and acquired immunity (mediated by T lymphocytes) [1].

MicroRNAs (miRNAs) are short, endogenous, non-protein-coding RNAs with important roles in health and disease. miRNAs negatively regulate gene expression by binding to the 3′-untranslated regions of mRNAs and initiating either translational repression or cleavage [2, 3].

Several studies have pointed to the involvement of miRNAs in the pathogenesis of psoriasis miR203, miR21, and miR146a that are all increased, whereas miR125b is downregulated compared with healthy skin. This suggests that miRNAs may play a role in psoriasis pathogenesis. Increased miR203 levels are associated with constitutive activation of STAT3 signaling, and this is achieved by direct targeting of SOCS3 for suppression, which is involved in inflammatory response and in keratinocyte functions [4]. So microRNA deregulation is involved in the pathogenesis of psoriasis and contributes to the dysfunction of the cross talk between resident and infiltrating cells [4].

These differentially expressed miRNAs are likely to influence many processes that are involved in psoriasis pathogenesis such as angiogenesis (miR21, miR31, and miR378), epidermal differentiation (miR135b, miR205, and miR203-AS), and inflammation (miR142-3p) [5]. Other miRNAs that are abnormally expressed in psoriasis are miR146a and miR125b. Overexpression of miR146a in psoriasis is controlled by the transcription factor NF-*κ*B, and the NF-*κ*B target genes TRAF6 and IRAK are involved in regulating the TNF-*α* signaling pathway [6], suggesting that miR146a may control TNF-*α* signaling in the skin [7]. In human T cells, miR146a is expressed at low levels in naïve T lymphocytes while it is abundantly expressed in memory T cells and it is induced upon TCR stimulation, consistent with its expression being dependent on NF-*κ*B induction [6, 8]. MiR146a has been shown both in vitro and in vivo to directly target two serine/threonine kinases, interleukin-1 receptor-associated kinase 1 (IRAK1) and tumor necrosis factor (TNF) receptor-associated factor 6 (TRAF6), that become associated with the interleukin-1 receptor (IL-1R) upon stimulation and are partially responsible for IL-1-induced upregulation of NF-*κ*B. This binding results in the suppression of the expression of NF-*κ*B's target genes such as the interleukins IL-6, IL-8, and IL-1*β*, and TNF-alpha (TNF-*α*) [9].

Narrow-band ultraviolet B (NB-UVB) therapy is associated with suppression of type I and type II IFN signaling, downmodulation of the Th17 pathway, and modulation of genes involved in epidermal differentiation in lesional psoriatic epidermis. In addition, several anti-inflammatory pathways, such as glucocorticoid, vitamin D, peroxisome proliferator activated receptor, and IL-4 signaling, are modulated by NB-UVB therapy [10].

Methotrexate is a successful and popular medicine used for treating severe psoriasis and some other serious or extensive skin conditions. It has anti-inflammatory properties; the precise mechanism of action is not understood, but it may relate to an increase in intracellular adenosine, a purine nucleoside that has anti-inflammatory effect. Methotrexate also has weak immune suppressive effects, also reducing the speed in which skin cells proliferate; it is a folate antagonist, which means it prevents the action of essential B vitamin, folic acid, on cellular function resulting in reduction in pyrimidine, purines, and methylation of DNA [11].

*Aim of Work*. The aim of this work is to compare the blood levels of miR146a in psoriasis patients and healthy controls and to evaluate the effect of NB-UVB and methotrexate treatment on blood miR146a levels in patients with psoriasis.

## 2. Patients and Methods

This case control study included forty patients with moderate or severe chronic plaque psoriasis who were selected from the outpatient Dermatology Clinic in Benha University Hospital, starting from January 2013 to October 2013. Patients had not received systemic immunosuppressive treatment or phototherapy, for at least 1 month, and topical therapy for 2 weeks before inclusion in this study. The control group consisted of 20 apparently healthy persons; both patients and control were between 18 and 65 years old. An informed consent was taken from the patients about their acceptance of being in the study, which was approved by Ethics Committee of Benha University.

A common questionnaire was used to complete demographic and characteristic information (e.g., age, sex, onset age, disease course, and family history). The Psoriasis Area and Severity Index (PASI) score of each psoriasis patient was evaluated.

The patients were divided into two groups. Group I had twenty patients with chronic plaque type psoriasis, 11 males and 9 females, with mean age 39.9 ± 16.9 years; they received methotrexate 12.5 mg Intramuscular once a week for 12 weeks.

Group II had twenty patients diagnosed with plaque-type psoriasis, 11 males and 9 females, with mean age 42.4 ± 14.1; their individual minimum erythema doses were assessed before the NB-UVB treatment. NB-UVB irradiation was administered to the whole body two to three times a week using a cabinet PCL 8000, Puva Combi Light, ARKADE, Heverlee, Belgium, equipped with fluorescent lamps UVB TL100W/01, Philips, Eindhoven, The Netherlands. About 24 sessions were given during a time of 2-3 months, with an initial dose of 0.1–0.3 J/cm^2^, increased accordingly depending on skin tolerance and clinical response.

### 2.1. Molecular Biology Investigations (Quantification of Human miRNA146a) by Real Time PCR (RT-PCR)

Blood samples from the patients were collected before and 12 weeks after the initiation of therapy with methotrexate and NB-UVB irradiation. The samples were collected immediately into RNA Protect Animal Blood tubes supplied by Qiagen, Germany. The tubes contain a reagent that lyses blood cells, designed for stabilization of intracellular RNA to preserve the gene expression profile. The samples were then stored at −80°C for further processing.

#### 2.1.1. Total RNA Extraction Including MicroRNA

Total RNA including miRNA was extracted from blood samples stabilized in RNA Protect Animal Blood tubes, using MiRNeasy Protect Animal Blood Kits (Qiagen, GmbH Hilden, Germany) according to manufacturer instructions [12].

#### 2.1.2. Reverse Transcription Step of Quantitative Real Time PCR

Total RNA was reverse transcribed into cDNA using specific reverse transcription (RT) primers (Table 1). The reaction mixture of 20 *µ*L contained 20 ng total RNA, 2 *µ*L of primer, 2 *µ*L of dNTPs, 1 *µ*L of RT enzyme, 2 *µ*L of RT buffer, 0.5 *µ*L (20 units) RNase inhibitor, and nuclease-free water up to 20 *µ*L. The cDNA was diluted to 200 *µ*L.

#### 2.1.3. Real Time PCR for Detection of miRNA146a

For miRNA quantity by real time PCR, the amount of the target miRNA (miRNA146a) was normalized against endogenous reference RNA. In this study we used U6 as normalization control. The relative quantities of the miRNA146a were normalized against the relative quantities of endogenous control (U6). Fold expression changes are calculated using the equation 2^−ΔΔct^ [13]. Real time PCR was performed using super real premix plus (sybr green) kit supplied by (TIANGEN, Biotech, Beijing). The reaction mix contained 10 *µ*L 2x super real premix plus, 1 *µ*L of forward primer, 1 *µ*L of reverse primer, 5 *µ*L of cDNA, 0.4 50x ROX reference dye, and 2.6 RNase-free water. Amplification of miRNA146a and U6 were done in separate PCR tubes.

### 2.2. Statistical Analysis

The clinical data were recorded on a report form. These data were tabulated and analyzed using the computer program SPSS (Statistical Package for Social Science) version 16 (*SPSS Inc., Chicago*) to obtain the following.

Descriptive statistics were calculated for the data in the form of (1) mean and standard deviation (±SD) for quantitative data; (2) frequency and distribution for qualitative data.

In the statistical comparison between the different groups, the significance of difference was tested using one of the following tests: (1) Student's *t*-test and Mann-Whitney test, used to compare mean of two groups of parametric and nonparametric quantitative data, respectively; (2) ANOVA test (*F* value), used to compare mean of more than two groups of quantitative data; (3) intergroup comparison of categorical data performed by using Fisher exact test (FET); (4) correlation coefficient, used to find relationships between variables.

## 3. Results

All the participants were examined and diagnosed by dermatologists. The mean age for group I was 39.9 ± 16.9 years, for group II was 42.4 ± 14.1 years, and was 44.6 ± 17.82 years for the controls (*P* = 0.677); the percentage of men was 55% for group I and for group II and 60.0% for the control (*P* = 0.15). The patients who have a family history of psoriasis are 30% in group I, 15% in group II (*P* = 0.02). The mean disease duration for group I was 2.9 ± 1.2 years, while it was 8 ± 5.6 years for group ΙΙ (*P* = 0.001). The mean value of PASI score before and after treatment was 26.5 ± 3.8, 9.7 ± 2.9, respectively, in group I (*P* = 0.001), while in group II it was 25.7 ± 2.8, 8.3 ± 1.9, respectively (*P* = 0.001).

**Figure 1:**
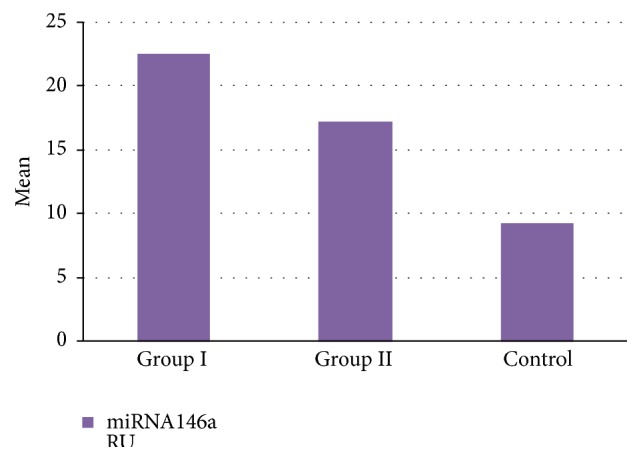
Comparison between group I, group II patients and control group as regards level of miRNA146a.

**Figure 2:**
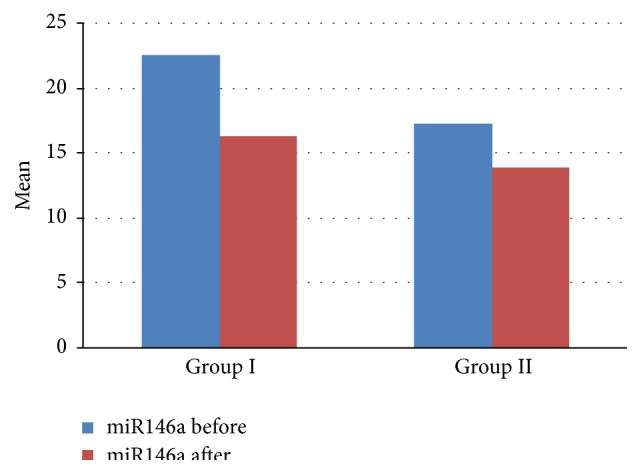
Comparison between group I and group II as regards level of miR146a before and after therapy.

The blood levels of miR146a in patients were significantly higher than control (Table 2 and Figure 1). The mean level of miRNA146a before and after therapy was 22.6 ± 3.3 RU, 16.3 ± 1.7 RU correspondingly (*P* = 0.001) in group I, while it was 17.3 ± 1.3, 14.0 ± 1.3 RU correspondingly (*P* = 0.002) in group ΙΙ (Table 3 and Figure 2).

**Figure 3:**
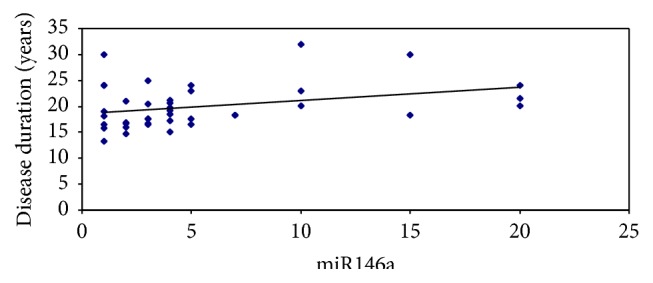
Correlation between miR146a level before treatment and disease duration among all patients.

There was no significant positive correlation between miR146a level before treatment and age and PASI score among all patients (*r* = 0.1, *P* value = 0.269) (*r* = 0.2, *P* value = 0.107), respectively. Whereas there was a significant positive correlation between miR146a and disease duration (*r* = 0.3, *P* value = 0.03) (Table 4 and Figure 3).

## 4. Discussion

Taganov et al. [6] reported that microRNA146a/b (miR146a/b) is induced in response to lipopolysaccharide (LPS) and proinflammatory mediators and that miR146a induction is regulated by NF-*κ*B. They also found that miR146a/b targets were TNF receptor-associated factor 6 (TRAF6) and IL-1 receptor-associated kinase 1 (IRAK1) genes and concluded that miR146 plays a role in fine-tuning innate immune responses by negative feedback, including downregulation of TRAF6 and IRAK1 genes. A recent major observation involving a new role for miRNAs is their ability to determine the efficacy of drugs. These findings gave rise to the field of miRNA pharmacogenomics [14].

Previous studies showed that most of tissue miRNAs could be detected in body fluid. Circulating miRNA in serum has been postulated as reliable biomarkers for disease prediction, diagnosis, and severity assessment. Under normal conditions, miRNAs are released mostly from circulating blood cells in serum, while under diseased conditions, miRNAs expression profiles are different and depend on the types and natures of the diseases [15].

The results of the present study showed that there were increased blood levels of miR146a in psoriatic patients higher than the control group. This was in agreement with the results of other studies [16–18]. miR146a is possibly involved in pathogenesis of psoriasis by modulating functions of macrophage [19], dendritic cells [20], Th1 cells [6], T reg cells [21], and Th17 cells [22]. Xia et al. [16] reported that increased miR146a with impaired function failed to repress the expression of* IRAK1*, thus inducing IL-17 persistence in psoriatic skin lesions.

In the current study, there was significant decrease of miR146a levels after therapy with methotrexate; this was in concurrence with the results of Pivarcsi et al. [17]; they found that anti-TNF-alpha therapy, etanercept, and methotrexate altered serum miRNA level in psoriatic patients. This may be due to the effect of methotrexate on DNA synthesis in epidermal cells and also due to its immunosuppressive effects on activated T cells that control psoriasis [23].

Also there was decrease in miR146a level in patients treated with NB-UVB in this study; this was in accordance with the results of Gu et al. [24]. This was due to the effect of NB-UVB leading to cell cycle arrest, T cell apoptosis, and apoptosis upon DNA damage as stated by Menter et al. [25] and Gu et al. [24].

There was no significant positive correlation between PASI score and the levels of correlation between miRNAs and PASI score, while there was a significant positive correlation between the disease duration and the levels of miR146a. This was in agreement of the results of Xia et al. [16]; this can be due to associated comorbidities including hypertension, atherosclerosis, and diabetes mellitus.

## 5. Conclusion

In conclusion, we have shown significant changes in blood levels of miR146a subsequent treatment with methotrexate and NB-UVB phototherapy, indicating considerable roles for these molecules in treatment of the disease. Early prediction of response to therapy using mRNA is an opportunity for effective individual medication and therefore allows preventing side effects by reducing the costs.

## Figures and Tables

**Table 1:** Primers used in the reverse transcription and PCR steps.

RNA	5′-3′ primer		
	Reverse transcription specific primers	PCR primers	
		Forward primer	Reverse primer
*miR146a*	*Stem loop primer* GTCGTATCCAGTGCGTGTCGTGGAGT CGGCAATTGCACTGGATACGACaaccca	GGGTGAGAACTGAATTCCA	CAGTGCGTGTCGTGGAGT

**Table 2:** Comparison between patient groups and control group as regards level of miRNA146a.

		Group I *N* = 20	Group II *N* = 20	Control *N* = 10	*F*-test	*P* value
miR146a level (RU)	Mean ± SD	(22.6 ± 3.3)	(17.3 ± 1.3)	(9.3 ± 3.8)	75.6	<0.001

**Table 3:** Comparison between patient groups as regards level of miR146a before and after therapy.

Variable	Group I *n* = 20 Mean ± SD	Group II *n* = 20 Mean ± SD	*t*-test	*P* value
miR146a (RU) before	(22.6 ± 3.3)	(17.3 ± 1.3)	6.6	<0.001
miR146a (RU) after	(16.3 ± 1.7)	(14 ± 1.3)	4.8	<0.001
*t*-test	7.6	8
*P* value	<0.001	<0.001

**Table 4:** Correlation between miR146a level before treatment and age, disease duration, and PASI score among all patients.

Variables	miR146a level	
	*r*	*P* value
Age	0.1	0.269
Duration of disease	0.3	0.03
PSAI score	0.2	0.107
