# Corrigendum to “Effect of Narrow-Band Ultraviolet B Phototherapy and Methotrexate on MicroRNA (146a) Levels in Blood of Psoriatic Patients”

**DOI:** 10.1155/2016/7168587

**Published:** 2016-11-09

**Authors:** Amal Abou El-Fadle, Asmaa M. Ele-Refaei, Fatma M. El-Esawy

**Affiliations:** ^1^Medical Biochemistry Department, Faculty of Medicine, Benha University, Benha, Egypt; ^2^Dermatology & Andrology Department, Faculty of Medicine, Benha University, Benha, Egypt

In the article titled “Effect of Narrow-Band Ultraviolet B Phototherapy and Methotrexate on MicroRNA (146a) Levels in Blood of Psoriatic Patients,” [1] Dr. Amal Abou El-Fadle was missing from the author list. The corrected author list is shown above. The “Acknowledgments” section was missing, and should be added as follows.
